# The global prevalence of postpartum psychosis: a systematic review

**DOI:** 10.1186/s12888-017-1427-7

**Published:** 2017-07-28

**Authors:** Rachel VanderKruik, Maria Barreix, Doris Chou, Tomas Allen, Lale Say, Lee S. Cohen, on behalf of the Maternal Morbidity Working Group, Jose Guilherme Cecatti, Sara Cottler, Olubukola Fawole, Veronique Filippi, Tabassum Firoz, Atf Ghérissi, Gathari Ndirangu Gichuhi, Gill Gyte, Michelle Hindin, Anoma Jayathilaka, Marge Koblinsky, Yacouba Kone, Nenad Kostanjsek, Isabelle Lange, Laura A. Magee, Arvind Mathur, Affette McCaw-Binns, Mark Morgan, Stephen Munjanja, Cihan Öztopcu, Elizabeth Sullivan, Özge Tunçalp, Peter von Dadelszen

**Affiliations:** 1 Department of Psychology and Neuroscience, University of Colorado, Boulder, 345 UCB Muenzinger, Office D314D, Boulder, CO 80309 USA; 2 UNDP/UNFPA/UNICEF/WHO/World Bank Special Programme of Research, Development and Research Training in Human Reproduction (HRP), Department of Reproductive Health and Research, World Health Organization, 20 Avenue Appia, 1211 Geneva, Switzerland; 3 Department of Strategy, Policy and Information (SPI), World Health Organization, 20 Avenue Appia, 1211 Geneva, Switzerland; 4 Center for Women’s Mental Health, Perinatal and Reproductive Psychiatry Clinical Research Program, Massachusetts General Hospital, Simches Research Building, 185 Cambridge St Suite 2200, Boston, MA 02114 USA

**Keywords:** Postpartum psychosis, Global prevalence, Systematic review

## Abstract

**Background:**

Mental health is a significant contributor to global burden of disease and the consequences of perinatal psychiatric morbidity can be substantial. We aimed to obtain global estimates of puerperal psychosis prevalence based on population-based samples and to understand how postpartum psychosis is assessed and captured among included studies.

**Methods:**

In June 2014, we searched PubMed, CiNAHL, EMBASE, PsycINFO, Sociological Collections, and Global Index Medicus for publications since the year 1990. Criteria for inclusion in the systematic review were: use of primary data relevant to pre-defined mental health conditions, specified dates of data collection, limited to data from 1990 onwards, sample size >200 and a clear description of methodology. Data were extracted from published peer reviewed articles.

**Results:**

The search yielded 24,273 publications, of which six studies met the criteria. Five studies reported incidence of puerperal psychosis (ranging from 0.89 to 2.6 in 1000 women) and one reported prevalence of psychosis (5 in 1000). Due to the heterogeneity of methodologies used across studies in definitions and assessments used to identify cases, data was not pooled to calculate a global estimate of risk.

**Conclusions:**

This review confirms the relatively low rate of puerperal psychosis; yet given the potential for serious consequences, this morbidity is significant from a global public health perspective. Further attention to consistent detection of puerperal psychosis can help provide appropriate treatment to prevent harmful consequences for both mother and baby.

**Electronic supplementary material:**

The online version of this article (doi:10.1186/s12888-017-1427-7) contains supplementary material, which is available to authorized users.

## Background

As a tragic but rare event, maternal mortality accounts for a small fraction of the overall burden of poor maternal health. Maternal morbidity, the health problems borne by women during pregnancy, childbirth and the postpartum period, also contributes to this burden in a major way. Measuring the burden of pregnancy and related post-partum morbidity is crucial to achieving the health and development goals articulated in the Sustainable Development Goals (SDGs) and those of the Global Strategy for Women’s, Children’s, and Adolescent health [1, 2]. The severity, occurrence, and timing of maternal conditions causing morbidity, and the measurement of their impact on women’s life, are key issues in the conceptualization of maternal morbidity.

To date, there has not been valid, routine, and comparable measurements of maternal morbidity [3]. To address this gap, the World Health Organization’s (WHO) Department of Reproductive Health and Research (RHR) introduced an initiative, led by a Maternal Morbidity Working Group (MMWG), composed of technical experts in maternal and women’s health, epidemiology, public health (including researchers and clinicians), program managers and consumer representatives, to estimate the burden of maternal morbidity based on existing evidence, and develop standard measures for maternal morbidity [4], in order to reflect the full continuum of maternal morbidity, including non-severe morbidity [5]. Several systematic reviews are being conducted as part of this initiative, including reviews on the prevalence of conditions included in the definition of maternal morbidity. The systematic reviews aim to provide recent epidemiological evidence for maternal conditions of high priority to support the implementation of maternal and neonatal health programs. As part of this effort, the MMWG recognized maternal mental health as a key area of study. This specific review will focus on the global prevalence of postpartum psychosis for reasons discussed further below.

Mental health is a significant contributor to global burden of disease. WHO estimates that for women of reproductive age (15–49), mental and behavioral disorders accounted for approximately 64 million global Disability Adjusted Life Years (DALYs) lost between 2000 and 2012 [6]. This burden also appears to be increasing over time. Between 2000 to 2012, mental and behavioral disorders increased from 5.9% to 7.3% as a proportion of all-cause DALYs [6]. Together these conditions are the fifth leading disorder category of DALYs and the leading global cause of all non-fatal burden of disease [6, 7]. Across all regions, girls and women are disproportionately affected compared to boys or men; the prevalence of depression among women is approximately two times greater than among men [8]. The highest proportion of DALYs for women occurs during key reproductive-age years. These findings highlight the importance of consideration for perinatal (i.e. timeframe just before and after birth) psychiatric morbidity. On average, 10% of pregnant women and 13% of postpartum women experience some type of mental disorder, most commonly depression or anxiety [9–11]. Prevalence rates have been found to be even greater in low and middle-income countries (LMICs), with average prevalence of about 16% antenatally and almost 20% postnatally [9].

The consequences of perinatal psychiatric morbidity can be substantial. Perinatal mental disorders are also associated with maternal complications and an increased risk of adverse neonatal and developmental outcomes for the child [12]. The severity of consequences can be heightened in the presence of comorbidities for the mother [13]; and comorbidities are increasing in pregnancy [14]. A recent systematic review focused on prevalence of common, *non-psychotic* perinatal mental disorders in low and middle-income countries (LMICs) [9]. Due to the potentially detrimental consequences of psychosis during the perinatal period, a systematic review on the prevalence of perinatal psychosis is warranted.

The current psychiatric nosology in the Diagnostic and Statistical Manual of Mental Disorders (DSM-5) does not recognize postpartum psychosis as a distinct disorder; rather, if a woman meets criteria for a brief psychotic disorder, the DSM-5 suggests adding “with postpartum onset” as a specifier if the onset is during pregnancy or within 4 weeks postpartum [15]. Some clinicians believe that the timeframe for the postpartum specifier should be extended to 6 months after delivery based on clinical experience suggesting that episodes can present beyond the 4 weeks [15]. As psychosis during the perinatal period typically occurs within the first 4 weeks of the postpartum period [16], this review will focus on *postpartum* psychosis as opposed to *perinatal* (which generally includes the ante- and intra-partum periods) psychosis more broadly.

Clinical features of postpartum psychosis include elated, dysphoric or labile mood, agitation, bizarre or disorganized behavior and thought processes, and insomnia, while psychotic symptoms can often include mood-incongruent delusions, hallucinations, or delusions of control, with content often related to the infant or self (e.g., harm to the infant or self) [15]. Though postpartum psychosis is relatively uncommon when compared to other mental disorders, the acuity and gravity of its consequences such as suicide or infanticide warrants specific attention [17]. Postpartum psychosis can increase risk for future non-pregnancy related psychotic episodes [18] and is a critical indicator of an underlying diagnosis of bipolar disorder [16, 19]. Improved detection of puerperal psychosis, a likely first indicator of an underlying diagnosis of bipolar disorder, could lead to proper diagnosis and treatment of bipolar disorder for perinatal women [20, 21].

Although a variety of evidence based treatment options exist for perinatal women, the rate of treatment for mental health disorders among perinatal women is low [22]. For example, less than 15% of women in an obstetrics clinic who scored above a cut-off for depression reported receiving any formal treatment [23]. Barriers to treatment can include factors such as limited resources or insurance to cover costs of treatment, time demands, transportation, stigma associated with treatment, or lack of knowledge regarding where to seek treatment [22, 24]. The treatment gap for mental health is significant, particularly in developing countries [25]. A first step towards addressing the treatment gap will be appropriate screening and monitoring of global perinatal mental health. In order to accurately monitor a given disorder, robust estimates of prevalence are needed, which this current systematic review will address.

Few reviews on the prevalence of common mental disorders in the perinatal period have focused on rare or more severe mental disorders, such as psychosis, on a global scale. The most frequently cited prevalence of puerperal psychosis is 1–2 per 1000 childbirths [26]. However, this estimate was determined in a developed country setting (Edinburgh in 1987) and is potentially out of date and not generalizable. The purpose of this systematic review is thus to obtain up-to-date global estimates of puerperal psychosis prevalence based on population-based samples and to understand how postpartum psychosis is being assessed and captured among the included studies.

## Methods

The protocol for all the systematic reviews associated with the maternal morbidity initiative was adapted from the peer-reviewed methodology established for the WHO Systematic Review of Maternal Morbidity and Mortality [27]. Both the search strategy and inclusion criteria, which have been described elsewhere, were tailored for the purposes of this review [27, 28]. Data for this study were identified by searches of PubMed, CiNAHL, EMBASE, PsycINFO, Sociological Collections, and Global Index Medicus and references from relevant articles with the search terms including “pregnancy”, “mothers”, “postpartum”, “epidemiologic methods”, “mood disorders”, “anxiety disorders”, “psychosis”, and “schizophrenia”. Search terms were modified for each of the databases by a librarian at the WHO headquarters in Geneva, Switzerland. See Additional file 1 for example search strategy of one database. We focused on publications irrespective of language since the year 1990 and the search was conducted in June 2014. Criteria for inclusion of studies in the review were: inclusion of primary data relevant to pre-defined mental health conditions, specified dates for data collection period, limited to data from 1990 onwards, sample size >200 and a clear description of methodology. While the onset of postpartum psychosis typically occurs within the first month after delivery, there is inconsistency in the field of what constitutes the postpartum timeframe for maternal morbidities [29], so the search included any articles referencing the “postpartum” period. All appropriate study designs (i.e., cross-sectional, cohort/longitudinal, controlled trial, incidence/prevalence survey, case-control) were included; for case-control studies, the sample size of the cases arm had to be over 200. Additionally, we sought summary estimates and not individual level patient data.

Over 24,000 reports were screened (by RV & MB) initially by titles and/or abstracts of which almost 23,000 were excluded upon initial title and/or abstract screen. Of the remaining approximately 1000 reports, almost 700 were included upon rescreening (by RV &MB) to be retrieved for full-text review. In a 2-step process, the same two reviewers independently determined whether studies met inclusion criteria. In step 1, each reviewer assessed and categorized abstracts of articles as “included,” “unsure,” or “excluded.” Discrepancies were resolved by consensus; a third reviewer (DC) adjudicated unresolved disputes and the judgment of this third reviewer was considered final. Based on title and abstract, we then categorized articles by conditions covered in the study (e.g. depression, anxiety, psychosis, etc.). For the purposes of this review, we focused on articles that included psychosis prevalence or incidence data as discussed in the introduction. If the conditions discussed in a given article were unclear or unstated, the full text was pulled to determine whether or not there was psychosis prevalence data. Efforts to pull all full text articles were made in conjunction with the WHO librarian and diverse WHO Headquarter staff fluent in the article languages, and for translation to relevant languages were made when possible.

As an effort to ensure that all relevant studies were captured for our review, we additionally conducted a science citation index search on the previously mentioned, keystone article by Kendell et al. [26] that has been the primary reference for prevalence of puerperal psychosis since its publication. A total of 566 citations were identified using Web of Science. We exported titles and abstracts for these articles and conducted a screening as described above to identify any articles meeting criteria for inclusion and/or data extraction that required full text review. Despite our efforts to include all articles regardless of location and language of publication, there may be a risk of publication bias in the studies identified and included in our review, in that studies written in a certain language or reporting certain findings may be more likely to be published than others.

**Fig. 1:**
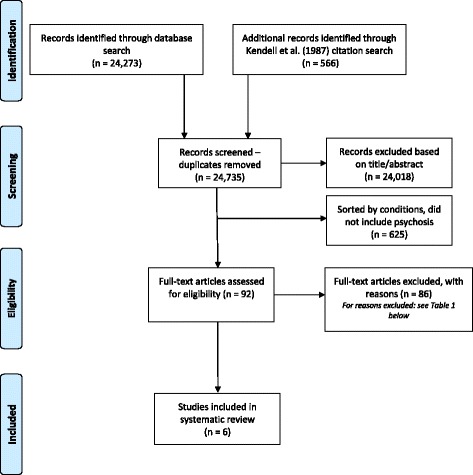
PRISMA Flow Diagram. From: Moher et al. [46]

The selection process was repeated until all articles were ultimately categorized as included or excluded. Screening title and abstracts, 92 were identified to report on psychosis and pulled for full text review. Following full-text review, 6 articles were included for this systematic review (see Table 1 for eligibility exclusion reasons). See Fig. 1 for flow of the article search, screening, and review, following PRISMA guidelines (and see Additional file 2 for the PRISMA Checklist).

**Table 1:** Breakdown for Exclusion Reasons at “Eligibility” Stage

Exclusion Reason	Number of Articles Excluded
No psychosis prevalence data	41
Review or editorial (i.e. not empirical study)	16
Duplicate article	8
Sample size <200	6
Not general population	5
Dates of data collection/publication	4
Not able to access article	4
Other	2
TOTAL	86

A data extraction instrument was used to obtain data from included studies. This instrument is composed of 40 items distributed in five modules, three of which were relevant to this review. Modules were designed to collect information on (i) the general study level characteristics such as design, population, setting, (ii) prevalence/incidence of psychological conditions, and (iii) quality assessment of reports. Reporting of definitions and of the procedures used for identification of cases was part of quality assessment. For quality appraisal, we extracted information on study design, sampling method, sources of data, completeness of follow-up or records, reported definitions and diagnostic procedures regarding outcomes. The evaluation of methodological and reporting quality was used to assess the reliability and accuracy of the data as objectively as possible, and has been described elsewhere [27]. Each paper was assessed for quality adequacy on four criteria and rated on a score of 1 (high – all four criteria are adequate) to 3 (low – none or only one criterion is adequate). Both RV and MB conducted separate quality reviews of each of the 6 articles; any differences were discussed and a final decision was made. (Please see Additional file 3 for complete quality assessment criteria.)

The same two reviewers (RV & MB) independently extracted data from each article using the data extraction form. See Additional file 4 for data extraction form used. A third party (DC) resolved disagreement in the same manner as for study inclusion. Given the diversity in the study design, measurement tools and definitions used, a meta-analysis of the findings was not performed. Below, we describe the included studies with an emphasis on the prevalence reported and criteria for identification of the cases. While the aim of our search was to capture prevalence data, as mentioned in the background, the majority of included studies reported on incidence of puerperal psychosis.

The funder of the study had no role in study design, data collection, data analysis, data interpretation, or writing of the report. The corresponding author had full access to all the data in the study and had final responsibility for the decision to submit for publication.

## Results

The systematic search yielded a total of six studies that met the screening criteria described above (see Table 2). Three of the studies involved reviews of national birth registries (Nager, Terp, Valdimarsdóttir), while a fourth employed data from a nationally representative household-level survey (Vesga-Lopez). These studies were conducted in Sweden (Nager and Valdmirsdóttir), Denmark (Terp) and the United States (Vesga-Lopez). As for the remaining two studies, one was conducted at a tertiary teaching institution in Ogun state, Nigeria and the other, at the community level in Maharashtra state, India, (Adefuye and Bang, respectively).

**Table 2:** Included Studies

	POPULATION	STUDY TYPE	DATES	NUMBER OF CASES	CONTROLS/DENOMINATOR	INCIDENCE (%)	SAMPLING
Adefuye et al. [31]	Post-partum women aged 19–43 years	Retrospective cohort study	1988–2007	23	9085	0.25% or 2.5 in 1000	All patients who developed mental disorders and were managed at Olabisi Onabanjo University Teaching Hospital
Bang et al. [34]	Pregnant and post-partum women aged (exact age range unknown)	Prospective observational study	1995–1996	2	772	0.26% or 2.6 in 1000	Women from catchment area of 39 intervention villages who became pregnant and were observed at home by trained village health workers, validated by physician
Nager et al. [44]	Primiparous women aged (exact age range unknown)	Retrospective cohort study	1975–2003	1413	1133,368	0.12% or 1.2 in 1000	All first-time mothers admitted to hospital due to psychiatric disorders up to 3 months after delivery (based on national registries)
Terp et al. [45]	Postpartum women aged years (exact age range unknown)	Incidence/Prevalence Survey	1973–1993	1133	1,270,117	0.089% or 0.89 in 1000	All women who delivered in Denmark and were admitted to hospital and diagnosed with psychosis up to 3 months after delivery (based on national registries)
Valdimarsdóttir et al. [33]	Primiparous women aged (exact age range unknown)	Incidence/Prevalence Survey	1983–2000	892	745,596	0.12% or 1.2 in 1000	All first-time mothers who were diagnosed with psychosis up to 3 months post-partum (based on national registries)
						PREVALENCE	
Vesga-Lopez et al. [32]	Postpartum women aged 18–50 years	Incidence/Prevalence Survey	2001–2002	(Exact number not reported - % only)	994	0.5% or 5 in 1000	Nationally representative sample of women interviewed during the 2001–2002 National Epidemiologic Survey on Alcohol and Related Conditions.

Four of the studies employed retrospective methods extracting information from the national birth registry (Valdimarsdóttir), national birth and psychiatric registries (Terp), national birth and hospital discharge registers (Nager), or women’s medical records (Adefuye) in order to calculate incidence. Vesga-Lopez utilized data from the United States National Epidemiologic Survey on Alcohol and Related Conditions survey, which gathered data from face-to-face interviews conducted in households across all 50 states and the District of Columbia to estimate prevalence. Bang et al. describes the only observational study that met the inclusion criteria, whereby community health workers visited a woman at home and assessed her condition (a physician validated the information and diagnosis was made by a computer program). One other study (Okano, 1998) fit our criteria [30], however it was not included in this review due to unclear methodology for prevalence calculations. Additionally, various studies presented above collected data prior to 1990; however in each case, authors were contacted and did not respond to email queries about either calculation methods nor data disaggregation, respectively. Given that the articles without disaggregated data were of high quality, they were included, although the Okano article was not included as the authors found the lack of transparency in calculations a major flaw.

Three studies, Nager, Terp and Valdimarsdóttir, extracted the women’s psychosis diagnoses from her records, as completed by a medical professional in national birth registries using International Classification of Diseases (ICD) coding. Terp also used admission information from hospital records from the Danish Psychiatric Central Register while Nager employed data from the Swedish national discharge register, both of which applied ICD codes for diagnosis. The Vesga-Lopez study, which drew from the national health survey of households in the United States, asked women to indicate whether a doctor or other medical professional had ever diagnosed her with schizophrenia or a psychotic disorder. Adefuye and Bang used women’s medical records or community health workers making home visits, respectively, to attain a diagnosis; the studies fail to detail the coding or diagnosis instruments used by the medical professional or community health workers to assess the woman’s psychotic state.

Most included studies reported on incidence (*n* = 5) and one study reported prevalence rates. Puerperal psychosis prevalence or incidence was assessed at various perinatal timeframes across the included studies. Most studies included only the postpartum period, but this timeframe varied across studies from a 2–32 day range [31], to up to a year postpartum [32]. One study compared incidence rates between two different postpartum time periods, breaking postpartum psychosis incidence down into two categories: within 90 days postpartum and after 90 days postpartum [33]. One study also included the pregnancy time period [34], although both of the psychosis cases reported in this study occurred during the puerperium period, and were associated with adverse perinatal outcomes.

The reported incidence of perinatal psychosis among our included studies ranged from 0.89 to 2.6 in 1000 women, aligning with the commonly reported prevalence rate of 1–2 per 1000 [26]. Terp et al. reported the lowest incidence (0.89 in 1000) in the study that had the greatest sample size (over 1.25 million), and assessed the time period of within 3 months postpartum. Of the six included studies in this review, Vesga-Lopez (2008) was the only study that reported prevalence of postpartum psychosis (5 in 1000). It is worth mentioning that the cases of psychotic disorder for this study were self-reported (“psychotic disorders were indicated by asking the respondent if she was ever told by a doctor or other health professional that she had schizophrenia or a psychotic disorder”). Additionally, the timeframe covered the entire year postpartum, which may also factor into the higher prevalence found. The sample size for Vesga-Lopez (2008) was the second smallest of our included studies, including 994 women. The study that reported the highest incidence rate (2.6 in 1000) was from Bang et al. [34], which the smallest sample size of our included studies (*N* = 772). The prevalence estimate was higher than any of the incidence estimates. Among the incidence studies, the highest estimates were among those studies with smaller sample sizes. However, given the limited number of studies included in this review, we are not able to draw a strong conclusion on how estimates differ by methodologies or sample sizes.

## Discussion

Our review found that post-partum psychosis incidence ranged between 0.89 and 2.6 in 1000 births across several countries, and one study reported prevalence to be 5 in 1000 births. These incidence estimates appear to be relatively consistent with the frequently cited prevalence of 1–2 in 1000 births for postpartum psychosis in the general population [26]. However, due to the wide variation in definitions and assessments used to capture cases of postpartum psychosis, it was not possible to pool data and make a summary estimate. There was inconsistency with instruments and methods used, as well as differing timeframes for assessment, to capture and identify postpartum psychosis. Such variation presents a challenge for obtaining an accurate estimate of the global burden of puerperal psychosis or to compare estimates among countries. Because of the varying methods of case identification criteria, as well as the limited number of studies that met our inclusion criteria, it is difficult to make cross-country comparisons of puerperal psychosis estimates. We cannot conclude that the incidence of postpartum psychosis is higher in studies conducted in less developed country settings, as there is not sufficient variability in the types of countries included in our review.

Furthermore, these findings highlight the lack of a concrete and consistent timeframe used to assess puerperal psychosis in the literature. By definition, postpartum psychosis occurs very shortly after birth, usually within days but up to a month postpartum [35]. However, several of the included studies assessed for psychosis well beyond this postpartum period, thus raising the question of whether or not these were true cases of puerperal psychosis.

Our search methodology includes some limitations that are important to consider when drawing conclusions from this study. First, we conducted a search of literature published within the past 15 years according to established WHO systematic review guidelines with the aim of including only the most recent studies. However, this excluded a number of rigorously conducted studies on puerperal psychosis that were published prior to this date [19, 26, 36]. Secondly, as we were interested in population-level estimates, and due to previously reported rare occurrence of postpartum psychosis, we only included studies that had a sample sizes of greater than 200. Thus, a number of studies were excluded due to small sample size. Although appropriate sample sizes for systematic reviews have been debated, there is evidence that small size studies are less likely to be published and inclusion of small studies may consequently introduce effects of publication bias [37]. Additionally, while Adefuye et al. present hospital based data, we felt it important to include given that while not representative of the entire Nigerian population, it represents data that are rare to find in the African region. The manuscript by Bang et al., though not representative of the entire Bangladeshi population includes a large and diverse catchment area, and again represents the only data found for the Asian region. Another limitation is that we did not assess the time of onset for the cases of postpartum psychosis across all included studies. A clinical feature of postpartum psychosis is rapid onset within the first month of delivery [15], yet some of the included studies considered the postpartum period to be up to 1 year after delivery. Thus, the estimates of postpartum psychosis in this review may reflect cases of onset beyond the first month following delivery.

A key strength of our systematic review is its comprehensive search strategy with over 24,000 papers screened. As part of the larger initiative to measure the burden of specific maternal morbidities, in order to follow the protocol for systematic reviews, we focused on published peer-reviewed articles with primary data collection. While it is a limitation that we did not carry out a grey literature search, we did attempt to expand our scope through the additional citation search of the keystone article by Kendell et al. [26]. Additionally, although we assessed quality of the studies, all publications relevant to our study aim in this review were included if they met inclusion criteria. Therefore, a bias may have been introduced in reporting findings in the studies of poor quality.

While this review focused on estimates among a general population, prevalence among at-risk populations may be greater and also clinically meaningful. For example, one study on teenagers found 2.5% prevalence [38] and another found a significantly higher rate (15%) for women with history of major affective disorder [39]. Such higher risk populations were outside of the scope of this current review but highlight that additional attention may be needed not just to pregnant women but also to pregnant women who are identified as at-risk.

There may be further cultural aspects of the prevalence and identification of puerperal psychosis that were beyond the scope of this review, but remain an important area for further investigation. For example, the aforementioned Okano et al. [30] study reported a low rate of puerperal psychosis. The authors suggested that there may be social and cultural factors that underlie lower rates of perinatal psychiatric illness in Japan [30]; citing the Japanese custom known as *Satogaeri bunben*, where the mother returns to her parental home for up to 2 months postpartum, as a factor in significantly reducing stress post childbirth. Alternatively, some believe that the relatively high stigma associated with mental illness in Japan may result in lower rates of diagnosis [40]. An area for future research that was beyond the scope of this review is with regards to how the presentation and identification of puerperal psychosis and its related stigma may differ across cultural contexts, and implications for measuring prevalence.

Although this systematic review confirms the relatively low rate of puerperal psychosis, given the potential for serious consequences associated with puerperal psychosis, even a low rate becomes a significant public health issue from a global perspective. As discussed previously, there can be tragic consequences such as suicide or infanticide [17]. Suicide has been identified as a leading cause of maternal death in some developed countries [41] and is also a leading cause of death in reproductive-aged women in the world’s two most populous countries, India and China [42]. For example, considering a country such as China where there are approximately 16 million births a year, there could potentially be up to 32,000 cases of puerperal psychosis within that same time period in just this country alone, at the incidence rate of 2 cases per 1000 childbirths among the general population.

Postpartum psychosis can also serve as a valuable first indicator of an underlying diagnosis of bipolar disorder [16] and can increase risk for future non-pregnancy related psychotic episodes [18]. The highest lifetime risk for first onset and recurrent episodes of bipolar disorder has been found in the postpartum period [18, 26]. Further attention to the detection and treatment of puerperal psychosis can help provide the woman with appropriate treatment that may prevent the possible detrimental consequences for both mother and infant associated with puerperal psychosis.

## Conclusions

This review confirms the relatively low rate of puerperal psychosis; yet the paucity of studies that meet our eligibility criteria highlights the critical gap in knowledge of puerperal psychosis prevalence from large-scale studies worldwide. As described above, postpartum psychosis is a key marker for the risk of future affective disorder, which is significant contributor to the global burden of disease [7, 43]. We recommend that further attention be given to identifying puerperal psychosis and monitoring incidence more consistently on a global scale. Appropriate detection of puerperal psychosis is needed to increase chances that a women will receive adequate treatment, which could help to mitigate the global disease burden and improve maternal and newborn health.

## Additional files

Additional file 1: Example Search Strategy for EMBASE. (DOCX 23 kb)

Additional file 2: PRISMA Checklist. (DOC 63 kb)

Additional file 3: Quality Assessment Criteria. (DOC 61 kb)

Additional file 4: Data Extraction Form. (DOCX 79 kb)

## References

[1] World Health Organization. Trends in Maternal Mortality: 1990 to 2013. Estimates by WHO, UNICEF, UNFPA, The World Bank and the United Nations Population Division; 2014.

[2] United Nations Secretary-General (2015). The Global Strategy for Women’s, Children’s and Adolescents’ Health (2016-2030).

[3] Vanderkruik RC, Tuncalp O, Chou D, Say L (2013). Framing maternal morbidity: WHO scoping exercise. BMC Pregnancy Childbirth.

[4] Say L, Barreix M, Chou D, Tunçalp Ö, Cottler S, McCaw-Binns A, Gichuhi GN, Taulo F, Hindin M (2016). Maternal morbidity measurement tool pilot: study protocol. Reprod Health.

[5] Firoz T, Chou D, von Dadelszen P, Agrawal P, Vanderkruik R, Tuncalp O, Magee LA, van Den Broek N, Say L (2013). Maternal morbidity working G: **measuring maternal health: focus on maternal morbidity**. Bull World Health Organ.

[6] World Health Organization. Health statistics and information systems. Disease and injury regional estimates, 2000–2012. Retrieved from: http://www.who.int/healthinfo/global_burden_disease/estimates_regional_2000_2012/en/.

[7] Whiteford HA, Degenhardt L, Rehm J, Baxter AJ, Ferrari AJ, Erskine HE, Charlson FJ, Norman RE, Flaxman AD, Johns N (2013). Global burden of disease attributable to mental and substance use disorders: findings from the global burden of disease study 2010. Lancet.

[8] Kessler RC, Berglund P, Demler O, Jin R, Merikangas KR, Walters EE (2005). Lifetime prevalence and age-of-onset distributions of DSM-IV disorders in the National Comorbidity Survey Replication. Arch Gen Psychiatry.

[9] Fisher J (2012). Cabral de Mello M, Patel V, Rahman a, Tran T, Holton S, Holmes W: **prevalence and determinants of common perinatal mental disorders in women in low- and lower-middle-income countries: a systematic review**. Bull World Health Organ.

[10] O'hara MW, Swain AM (1996). Rates and risk of postpartum depression-a meta-analysis. Int Rev Psychiatry.

[11] Hendrick V, Altshuler L, Cohen L, Stowe Z (1998). Evaluation of mental health and depression during pregnancy: position paper. Psychopharmacol Bull.

[12] Field T (2011). Prenatal depression effects on early development: a review. Infant Behav Dev.

[13] Howard LM, Piot P, Stein A (2014). No health without perinatal mental health. Lancet.

[14] Souza J, Tuncalp O, Vogel J, Bohren M, Widmer M, Oladapo O (2014). Obstetric transition: the pathway towards ending preventable maternal deaths. BJOG.

[15] Monzon C, Lanza di Scalea T, Pearlstein T. Postpartum psychosis: updates and clinical issues. Psychiatric times; 2014.

[16] Sit D, Rothschild AJ, Wisner KL (2006). A Review of Postpartum Psychosis. J Women's Health..

[17] Jones I, Chandra PS, Dazzan P, Howard LM (2014). Bipolar disorder, affective psychosis, and schizophrenia in pregnancy and the post-partum period. Lancet.

[18] Munk-Olsen T, Laursen TM, Pedersen CB, Mors O, Mortensen PB (2006). New Parents and Mental Disorders A Population-Based Register Study. JAMA Psychiatry.

[19] Reich T, Winokur G (1970). Postpartum psychoses in patients with manic depressive disease. J Nerv Ment Dis.

[20] Spinelli MG (2005). Infanticide: contrasting views. Arch Womens Ment Health.

[21] Spinelli MG (2009). Postpartum Psychosis: Detection of Risk and Management. Am J Psychiatry.

[22] Ko JY, Farr SL, Dietz PM, Robbins CL (2005). Depression and treatment among U.S. pregnant and nonpregnant women of reproductive age. J Womens Health (Larchmt) 2012.

[23] Marcus SM, Flynn HA, Blow FC, Barry KL (2003). Depressive Symptoms among Pregnant Women Screened in Obstetrics Settings. J Women's Health.

[24] O'Mahen HA, Flynn HA (2008). Preferences and perceived barriers to treatment for depression during the perinatal period. J Women's Health (Larchmt).

[25] Kohn R, Saxena S, Levav I, Saraceno B (2004). The treatment gap in mental health care. Bull World Health Organ.

[26] Kendell RE, Chalmers JC, Platz C (1987). Epidemiology of puerperal psychoses. Br J Psychiatry..

[27] Gulmezoglu AM, Say L, Betran AP, Villar J, Piaggio G (2004). WHO systematic review of maternal mortality and morbidity: methodological issues and challenges. BMC Med Res Methodol.

[28] Say L, Pattinson RC, Gulmezoglu AM (2004). WHO systematic review of maternal morbidity and mortality: the prevalence of severe acute maternal morbidity (near miss). Reprod Health.

[29] Vanderkruik R, Tuncalp O, Chou D, Say L. Framing maternal morbidity: WHO scoping exercise. BMC Pregnancy Childbirth. 2013;13(213). 10.1186/1471-2393-13-213 PMC3840647 24252359

[30] Okano T, Kumar NR, Kaneko E, Tamaki I, Hanafusa I, Hayashi M, Matsuyama A (1998). An epidemiological and clinical investigation of postpartum illness in Japanese mothers. J Affect Disord.

[31] Adefuye PO, Fakoya TA, Odusoga OL, Adefuye BO, Ogunsemi SO, Akindele RA. Post-partum mental disorders in Sagamu. East Afr Med J. 2008;85(12):607–11. 19413217

[32] Vesga-López O, Blanco C, Keyes K, Olfson M, Grant BF, Hasin DS. Psychiatric Disorders in Pregnant and Postpartum Women in the United States. Arch Gen Psychiatry. 2008;65(7):805–15. 10.1001/archpsyc.65.7.805 PMC2669282 18606953

[33] Valdimarsdóttir U, Hultman CM, Harlow B, Cnattingius S, Sparén P. Psychotic illness in first-time mothers with no previous psychiatric hospitalizations: a population-based study. PLoS Med. 2009 Feb 10;6(2):e13. 10.1371/journal.pmed.1000013 PMC2637917 19209952

[34] Bang RA, Bang AT, Reddy MH, Deshmukh MD, Baitule SB, Filippi V. Maternal morbidity during labour and the puerperium in rural homes and the need for medicalattention: A prospective observational study in Gadchiroli, India. BJOG. 2004;111(3):231–8. 10.1111/j.1471-0528.2004.00063.x 14961884

[35] American Psychiatric Association A (2000). Diagnostic and statistical manual of mental disorders: DSM-IV-TR.

[36] Protheroe C (1969). Puerperal psychosis: A long-term study 1927–1961. Br J Psychiatry.

[37] Slavin RE, Smith D. Effects of Sample Size on Effect Size in Systematic Reviews in Education. Paper presented at the annual meetings of the Society for Research on Effective Education. Crystal City; 2008. Retrieved from http://bestevidence.org.uk/assets/eff_sample_size_review_Mar_2008.pdf.

[38] Mitsuhiro SS, Chalem E, Moraes Barros MC, Guinsburg R, Laranjeira R (2009). Brief report: prevalence of psychiatric disorders in pregnant teenagers. J Adolesc.

[39] Di Florio A, Jones L, Forty L, Gordon-Smith K, Blackmore ER, Heron J, Craddock N, Jones I (2014). Mood disorders and parity - a clue to the aetiology of the postpartum trigger. J Affect Disord.

[40] Mino Y, Aoyama H, Froom J (1994). Depressive disorders in Japanese primary care patients. Fam Pract.

[41] Oates M (2003). Perinatal psychiatric disorders: a leading cause of maternal morbidity and mortality. Br Med Bull.

[42] Miranda J, Patel V (2005). Achieving the Millennium Development Goals: does mental health play a role?. PLoS Med.

[43] Whiteford HA, Ferrari AJ, Degenhardt L, Feigin V, Vos T (2015). The global burden of mental, neurological and substance use disorders: an analysis from the global burden of disease study 2010. PLoS One.

[44] Nager A, Sundquist K, Ramírez-León V, Johansson LM. Obstetric complications and postpartum psychosis: a follow-up study of 1.1 million first-timemothers between 1975 and 2003 in Sweden. Acta Psychiatr Scand. 2008;117(1):12–9. 10.1111/j.1600-0447.2007.01096.x 17941968

[45] Terp IM, Mortensen PB. Post-partum psychoses. Clinical diagnoses and relative risk of admission after parturition. Br J Psychiatry. 1998;172:521–6. 10.1192/bjp.172.6.521 9828994

[46] Moher D, Liberati A, Tetzlaff J, Altman DG, The PRISMA Group (2009). Preferred Reporting Items for Systematic Reviews and Meta-Analyses: The PRISMA Statement. PLoS Med 6(6):e1000097. doi:10.1371/journal.pmed1000097 PMC3090117 21603045

