# Supplementary material for: The global prevalence of postpartum psychosis: a systematic review
Source: BMC Psychiatry. 2017 Jul 28;17:272. doi: 10.1186/s12888-017-1427-7 (PMC5534064; doi:10.1186/s12888-017-1427-7)
Supplement: Supplementary file 1 — Example Search Strategy for EMBASE. (DOCX 23 kb) [file 12888_2017_1427_MOESM1_ESM.docx]

| **#** | **Searches** |
| --- | --- |
| 1 | 'pregnant woman'/exp OR  'pregnancy'/exp OR 'child bearing':ti,ab OR  'childbearing':ti,ab OR pregnant:ti,ab OR pregnancy:ti,ab OR  'puerperium'/exp OR 'post partum':ti,ab OR postpartum:ti,ab OR 'ante natal':ti,ab OR prenatal:ti,ab OR postnatal:ti,ab OR 'post natal':ti,ab |
| 2 | \| 'biostatistics'/exp OR 'comparative study'/exp OR 'controlled study'/exp OR 'treatment outcome'/exp OR 'epidemiology'/exp OR 'mathematical phenomena'/exp OR 'biostatistics'/exp OR 'epidemiological data'/exp OR mortality:ti,ab OR incidence:ti,ab OR prevalence:ti,ab OR ‘epidemiology’:lnk OR ‘health economics’/exp \| \| --- \| |
| 3 | 'neurosis'/exp OR 'anxiety disorder'/exp OR 'anxiolytic agent'/exp OR 'adjustment disorder'/exp OR 'somatoform disorder'/exp OR 'Munchausen syndrome'/exp OR anxieties:ti,ab OR agoraphobi*:ti,ab OR (phobic:ti,ab AND disorder*:ti,ab) OR (panic:ti,ab AND (disorder*:ti,ab OR panic*:ti,ab)) OR (obsessive:ti,ab AND compulsi*:ti,ab ) OR 'post-traumatic’:ti,ab OR PTSD:ti,ab OR (neurotic:ti,ab OR disorder*:ti,ab) OR (adjustement:ti,ab OR disorder*:ti,ab) OR (obsessive:ti,ab AND behavio*:ti,ab ) OR 'munchhausen disorder':ti,ab OR (somatoform:ti,ab AND disorder*:ti,ab) |
| 4 | 'mood disorder'/exp OR 'Mood Disorder Questionnaire'/exp OR 'mental disease'/exp OR (mood:ti,ab OR disorder*:ti,ab) OR suicide*:ti,ab OR manic:ti,ab OR hypomania:ti,ab OR biopolar:ti,ab |
| 5 | 'schizophrenia'/exp OR 'personality disorder'/exp OR 'psychosis'/exp OR Schizophren*:ti,ab OR (paranoid:ti,ab OR disorder*:ti,ab) OR (psychotic:ti,ab OR disorder*:ti,ab) OR schizotypal*:ti,ab OR (delusional:ti,ab OR disorder*:ti,ab) OR Schizoaffective:ti,ab OR psychosis:ti,ab |
