# Supplementary material for: The global prevalence of postpartum psychosis: a systematic review
Source: BMC Psychiatry. 2017 Jul 28;17:272. doi: 10.1186/s12888-017-1427-7 (PMC5534064; doi:10.1186/s12888-017-1427-7)
Supplement: Supplementary file 3 — Quality Assessment Criteria. (DOC 61 kb) [file 12888_2017_1427_MOESM3_ESM.doc]

| **First author** | | **Quality Score** | | |
| --- | --- | --- | --- | --- |
| Adefuye et al (2008) | | 2 Medium quality | | |
| Bang et al (2004) | | 3 Low quality | | |
| Nager et al (2008) | | 2 Medium quality | | |
| Terp et al (1998) | | 2 Medium quality | | |
| Valdimarsdóttir et al (2009) | | 1 High quality | | |
| Vesga-Lopez et al (2008) | | 1 High quality | | |
|  | | | | |
| **QUALITY ASSESSMENT** | **Adequate** | | | **Inadequate** |
| *Sampling* | (1) Random, consecutive, etc. | | | (2) Selected groups, etc. |
| *Description of population* | (1) Type and characteristics of population and type of setting are specified | | | (2) Either of them not specified |
| *Follow up/ completeness of data* | (1) Reported and <20% loss | | | (2) Not reported or >20% loss |
| *Definition of outcome/*  *description of diagnostic procedures* | (1) Definition and diagnostics reported | | | (2) Definition or diagnostics not reported |
| ***Decision*** | | | **(1) High quality if all four criteria are adequate**   1. **(2) Medium quality if two or three criteria are adequate** 2. **(3) Low quality if none or only one criterion is adequate** | |
