# Supplementary material for: The global prevalence of postpartum psychosis: a systematic review
Source: BMC Psychiatry. 2017 Jul 28;17:272. doi: 10.1186/s12888-017-1427-7 (PMC5534064; doi:10.1186/s12888-017-1427-7)
Supplement: Supplementary file 4 — Data Extraction Form. (DOCX 79 kb) [file 12888_2017_1427_MOESM4_ESM.docx]

WHO Maternal Morbidity – Mental Health Systematic Review

**MODULE 1**

| **#** | **Question** | **Response codes** | |
| --- | --- | --- | --- |
| 1 | RefID Number | # \|___\|___\|___\|___\| | |
| 2 | Name of data extractor |  | |
|  | **STUDY IDENTIFIERS** | | |
| 3 | Date of extraction (dd/mm/yy) | | \|___\|___\|___\|___\|___\|___\|  d d m m y y |
| 4 | Last name of the first author | |  |
| 5 | Name of country | |  |
| 6 | Is the study published? | | (1) YES (2) NO |
| 7 | Year of publication | |  |
| 8 | Language of paper | | (1) English  (2) French  (3) German  (4) Chinese  (5) Spanish  (6) Russian  (7) Portuguese  (8) Other: __________________________________________ |
| 9 | Region or sub-region of the study (for example: Northwest province, etc). | | Describe: |
| 10 | Study Period | | Month/Year Month/Year  \|____\|___\| \|____\|___\| to \|____\|___\| \|____\|___\| |
| 11 | Was the study population selected from: | | (1) National  (2) Province/Region  (3) City  (4) Medical facility  (5) Other  Specify_________________________ |
| 12 | Study design | | (1) Cross-sectional  (2) Cohort/Longitudinal  (3) Controlled Trial  (4) Incidence/Prevalence Survey  (5) Case-Control  (6) Unknown  (7) Other  Specify____________________________ |
| 13 | Sampling | | (1) Random sample  Specify the method of randomization  ______________________________  (2) Non-random sample  Specify the method of sampling  ______________________________  (3) Total population (i.e. census or all admissions)  (4) Unknown |
| 14 | Which category best describes the study population? | | (1) Rural  (2) Urban  (3) Periurban/slum  (4) Mixed  (5) Population not well described |
| 19 | Description of the characteristics of the population studied (eg. Socio-economic status, ethnic group, age group etc) | | 1. SES  - Low SES - Mixed SES - Other:  1. Race  - American Indian or Alaska Native - Asian - Black or African American - Native Hawaiian or Other Pacific Islander - White  1. Ethnicity  - Hispanic or Latino - Not Hispanic or Latino  1. Age group  - Teenagers - 20-30 - 31-35 - >36 - Mixed ages  1. Other   specify: ____________________ |
| 15 | Data Source | | (1) Medical Record  (2) Special Survey/Interview  (3) Multiple sources  (4) Clinical data collected for the study  (5) Other  Specify______________________________ |
| 16 | Lowest unit of data source | | (1) Cluster  Number of clusters_______  (2) Individual  (3) Other  Specify__________________________________ |
| 17 | Number of eligible subjects (if available) | |  |
| 18 | Sample size studied | |  |
| 20 | Description of the health characteristics of the population (e.g. healthy women, women with a specific condition etc) | | (1) Healthy Pregnant Women  (2) Healthy Postpartum Women  (3) Pregnant Women with a specific condition  - Condition: ________________  (4) Postpartum Women with a specific condition  - Condition: ________________  (5) Other: __________________ |
| 21 | Is there information regarding the proportion of study subjects lost to follow-up (or non-responders for surveys or those not included in the final analysis for cross-sectional designs)? | | (1) YES (2) NO (3) N/A |
| 22 | If **“Yes”,** percentage of the study subjects lost to follow-up (or non-responders for surveys or those not included in the final analysis for cross-sectional designs and RCTs) | | Of All screened + for morbidity:   - - _______(#) Followed up/received treatment   - _______(#) No treatment   State reason if provided: __________________   - - _______(#) LTF   - Total # screened: __________ |
| 23 | Have the characteristics of the study subjects lost to follow-up (or non-responders for surveys or those not included in the final analysis for cross-sectional designs and RCTs) been described? | | (1) YES (2) NO (3) NA |
| 24 | If **“Yes”,** are the characteristics of the study subjects lost to follow-up (or non-responders for surveys or those not included in the final analysis for cross-sectional designs and RCTs) different from the rest of the population? | | (1) YES (2) NO (3) NA  If yes, elaborate: _________________________________ |
| 25 | Place of delivery/abortion | | (1) Home  (2) Hospital  (3) Mixed  (4) Unknown  (5) Not applicable  (6) Other  Specify________________________________ |
| 26 | If applicable, description of the intervention | |  |
| 27 | Forms of reporting data | | (1) Crude  (2) Adjusted for confounding variables  (3) Crude and adjusted  (4) Standardized by population distribution  (5) Adjusted and standardized |
| 28 | Are there data regarding risk factors? | | (1) YES (2) NO  If yes, summarize data:   - History of violence: RR = ____________ - History of mental disorder: RR = ________ - Poverty: RR= _________________ - Etc…*get as we find them!* |
| 29 | Remarks for the characteristics of the study | |  |

**MODULE 2**

|  | **MATERNAL MORBIDITY – Common Perinatal Mental Disorders or Psychosis** | | | | | |  |
| --- | --- | --- | --- | --- | --- | --- | --- |
| 30 | **Prevalence/Incidence** | | | | | | **Instrument Used/** |
|  |  | (i) | (ii) | (iii)* | (iv) | (v)** |  |
|  | Maternal Morbidity  *If more than 1 condition, include all* | No of cases | Denominator | Type | Percentage | Timing |  |
|  |  |  |  |  |  |  |  |
|  |  |  |  |  |  |  |  |
|  |  |  |  |  |  |  |  |

*** Type: ** Timing of diagnosis:**

1. Live Births 1. Antepartum

2. Pregnancies 2. During delivery

3. Deliveries 3. Postpartum

4. Women 4. More than one of the above

5. Not specified

| 31 | Does the study include a definition for the morbidity? | (1) YES (2) NO |
| --- | --- | --- |
| 32 | If definition is included, please specify: |  |
| 33 | The criteria utilized to identify morbidity cases in the study | (1) Disease specific (received a diagnosis)  (2) Intervention specific (e.g. medication use)  (3) Organ-system based  (4) Mixed  5) Score on psychological instrument/scale:  Instrument: ______________________  Score cut off for “case” inclusion: ____________________ |
| 34 | Does the study explain the method of assessment for the morbidity? | (1) YES (2) NO |
| 35 | If YES, please specify: |  |

| **REMARKS FOR MATERNAL MORBIDITY:** |
| --- |
